# Maternal and Child Health of Internally Displaced Persons in Ukraine: A Qualitative Study

**DOI:** 10.3390/ijerph14010054

**Published:** 2017-01-09

**Authors:** Svitlana Nidzvetska, Jose M. Rodriguez-Llanes, Isabelle Aujoulat, Julita Gil Cuesta, Hannah Tappis, Joris A. F. van Loenhout, Debarati Guha-Sapir

**Affiliations:** 1 Centre for Research on the Epidemiology of Disasters, Institute of Health and Society, Université catholique de Louvain, Brussels 1200, Belgium; julita.gilcuesta@uclouvain.be (J.G.C.); joris.vanloenhout@uclouvain.be (J.A.F.v.L.); debarati.guha@uclouvain.be (D.G.-S.); 2 Food Security Unit, Sustainable Resources Directorate, European Commission Joint Research Centre, I-21027 Ispra, Italy; jose-manuel.rodriguez-llanes@jrc.ec.europa.eu; 3 Institute of Health and Society, Université catholique de Louvain, Brussels 1200, Belgium; isabelle.aujoulat@uclouvain.be; 4 Department of International Health, Johns Hopkins Bloomberg School of Public Health, Johns Hopkins University, Baltimore, MD 21205, USA; hannah.tappis@jhu.edu

**Keywords:** Ukraine, maternal health, child health, reproductive health, mental health, IDPs, conflict, crisis, humanitarian response

## Abstract

Due to the conflict that started in spring 2014 in Eastern Ukraine, a total of 1.75 million internally displaced persons (IDPs) fled the area and have been registered in government-controlled areas of the country. This paper explores perceived health, barriers to access to healthcare, caring practices, food security, and overall financial situation of mothers and young children displaced by the conflict in Ukraine. This is a qualitative study, which collected data through semi-structured in-depth interviews with nine IDP mothers via Skype and Viber with a convenience sample of participants selected through snowball technique. Contrary to the expectations, the perceived physical health of mothers and their children was found not to be affected by conflict and displacement, while psychological distress was often reported. A weak healthcare system, Ukraine’s proneness to informal payments, and heavy bureaucracy to register as an IDP were reported in our study. A precarious social safety net to IDP mothers in Ukraine, poor dietary diversity, and a generalized rupture of vaccine stocks, with halted or delayed vaccinations in children were identified. Increasing social allowances and their timely delivery to IDP mothers might be the most efficient policy measure to improve health and nutrition security. Reestablishment and sustainability of vaccine stocks in Ukraine is urgent to avoid the risks of a public health crisis. Offering psychological support for IDP mothers is recommended.

## 1. Introduction

As a result of the Euromaidan revolution started in November 2013, the former Ukrainian President Victor Yanukovich was removed from his position and forced into exile in February 2014. Soon after, the attitudes from citizens of Donetsk and Luhansk regions (strongly supporting the former president) towards the Ukrainian state quickly deteriorated, followed by riots against the new government. The new government reacted immediately and sent troops to control the protests. However, the troops met serious resistance, which escalated into the start of an armed conflict affecting these territories.

Active fights took place from April 2014 until a ceasefire was signed on 15 February 2015, with clashes continuing until today. The northern parts of the Donetsk and Luhansk regions are, at the time of writing, under control of the Ukrainian government. The International Committee of the Red Cross qualified the fighting in the east of Ukraine as a “non-international armed conflict” [1].

According to the Office for the Coordination of Humanitarian Affairs (OCHA) [2,3], as of June 2016, the ongoing conflict has caused 2008 civilians deaths, and the displacement within Ukraine of around 1.75 million people. Among them, almost 1.1 million are women [4]. The government provides poor financial assistance to Internally Displaced Persons (IDPs) as well as little legal support. An able-bodied IDP is entitled to EUR 16.2 of state support per month, whereas the average monthly rent of a single-room apartment without utilities starts at EUR 130 in the regions or EUR 185 in the capital city. IDP dependents such as children or pensioners are entitled to a monthly allowance of EUR 32.4. According to OCHA reports [2,3], these payments constitute the major or the only source of income for many IDPs. During the Ukrainian conflict, it was already noticed that IDPs were at higher risk of poor financial status [5], poor diet [6], shortages of vaccines [7], and disruption of breastfeeding practices [5].

Pregnant women, women with young children, and these children themselves are among the most vulnerable groups within populations affected by conflict [8]. In crisis settings, access to health services is limited, which raises the risk of newborn mortality [9]. Conflicts also seriously affect children’s health: most children do not die from weapons directly, but from the preventable diseases which become more prevalent due to destroyed health systems and infrastructure [8]. WHO and UNICEF [10] have reported severe disruptions of vaccination practices in Ukraine since 2014. Moreover, conflict and displacement also have significant harmful psychological impact on both mothers and children [11]. However, these issues are often overlooked by traditional humanitarian response measures, which tend to focus on the provision of basic services, such as food and water, shelter, sanitation, and medical emergency services [9,12]. 

Social, economic, and political transformation following Ukrainian independence in 1991 had a strong negative impact on the population’s health [13]. The current Ukrainian health care system yet functions using the Semashko model from the Soviet Union, and its services are de jure free to any Ukrainian citizen. However, the infrastructure is poor and the sector remains significantly under-financed which favours high prevalence of informal payments [13].

So far, the conflict in Ukraine has displaced more than 215,000 children internally [14]. Lack of access to health services, shortage of medicines, and poor living conditions are threatening disease outbreaks among children. According to UNICEF, access to employment, education, and health care represent further challenges to IDPs, and problems have emerged in the provision of food, clothing, and medicines to IDP children [14]. However, we could not identify any study that looked at the impact on maternal and child health in Ukraine from the perspective of young mothers.

We aim at describing the experiences of IDP mothers of children less than two years of age with regards to health during and after pregnancy, access to healthcare, food security, and finances.

## 2. Materials and Methods

### 2.1. Study Setting

According to the data by State Ukrainian Statistics Agency, Ukraine’s population accounted for 42.8 million permanent residents as of 1 September 2015. Ukraine still has the highest mortality rate in Europe with 14.7 deaths per 1000 citizens in 2014 [15]. Ukraine has a very high literacy rate of 99.7%, and its population is well-educated with a university enrolment ratio of 82% [16]. However, the possession of a degree does not guarantee a well-paid job. Ukrainian salaries are very low, and the situation has significantly deteriorated after the political events of 2013–2014, when the national currency dropped by a factor three. According to the State Statistics Service of Ukraine, the average monthly salary in the country was about EUR 179 at the moment of data collection (exchange rate fixed for this research as UAN 27.3 per EUR 1 as per 11 July 2016).

### 2.2. Interview Format

The primary data for this study were collected through semi-structured in-depth interviews with internally displaced mothers of children less than two years old in Ukraine (the research attempted to cover a large geographical area). The research instrument was developed based on the previously available literature on maternal and child health of internally displaced persons in general and in Ukraine in particular [17,18,19]. The multi-methods approach was used in survey design. The interview guide was predominantly composed by open questions (Appendix A), but for the perceived health theme respondents were additionally offered to rank their health and the health of their children on a scale from 0 to 100. These ranks were used to introduce the topic of health within the interview. The in-depth exploration of health status was then obtained by the qualitative interview guide used. The interview guide covered the following themes: demographic information, military and displacement experience, perceived health of mother and child, use of health care services, lactation, nutrition, and household financial situation. In this study, we used the WHO definition of health: “Health is a state of complete physical, mental and social well-being and not merely the absence of disease or infirmity” [20]. In addition, the participants were asked about demographic data such as age, employment status, occupation, household composition, place of origin, and place of displacement.

The interview guide was first designed in English, translated to Russian by the first author of the research, and then back-translated to English by an independent translator. 

### 2.3. Data Collection

All interviews were conducted in Russian via Skype or Viber and lasted between 20 and 40 min. Interviews were conducted from 1 May to 30 June 2016. According to the preferences of respondents, video-call was or was not used. This was intended to help respondents feel comfortable and secure during an interview [21]. All interviews were recorded with the help of a voice-recording tool. The intended number of respondents was not pre-determined [21]. 

### 2.4. Study Population

The interviewees were recruited via convenience sampling. There were two starting points of recruitment, based on the provided lists of mothers with children of eligible age. Assistance was given by two local NGOs: ZOVU, which is in contact with IDPs throughout the entire territory of Ukraine, and Vostok-SOS, operating in Kyiv, the capital. The author of the research had previously collaborated with these two NGOs in Ukraine. The eligibility criterion was to be an IDP mother of a child up to two years of age at the time of interview. The age limit for a child was set at two years, as it is known to be critical period for child development and survival [22]. Another motivation for setting this age limit was that in spring 2016, the period of data collection, the Ukrainian conflict reached its second year, meaning that respondents were either pregnant or gave birth to their child after displacement. The researchers were not able to provide any technical assistance, thus respondents had to use devices available to them at their place of location.

### 2.5. Data Analysis

Thematic analysis was used to examine the data from this study, and peer review was used as a major validation strategy [23]. This method requires researchers to develop a set of codes, domains, or themes and to subsequently categorize the data collected. This analysis involves a narrative approach, which is well suited to provide a rich and detailed account of the social formations shaping subjective experiences of health and well-being [24]. Another advantage of thematic analysis is that it allows analyzing data generated by both homogenous and heterogeneous samples and does not set a limit for the size of the dataset [23]. For data analysis, the research differentiated between two concepts related to perceived health: physical health, referring to physical well-being and the absence of major disease; and mental health, referring to social and emotional well-being.

### 2.6. Ethical Considerations

All respondents provided written informed consent prior to participation, sending its scanned version or picture by e-mail, and did not receive any incentives to participate in the study. The participants’ written agreements to record were obtained prior. Recordings were stored safely and kept confidential as per international ethical guidelines [25]. Ethical approval compliant with the Helsinki declaration [25] was obtained from the Ethics Committee of the Saint-Luc Faculty of Medicine, Université catholique de Louvain, Brussels, Belgium (No*.* B403201628963).

## 3. Results

Out of 16 women contacted, 9 women agreed to participate and had the necessary equipment for Skype/Viber call. Table 1 presents some key characteristics of the interviewed IDP mothers, including occupation, employment status, and family composition.

### 3.1. Perceived Health of IDP Mothers and Children

The majority of mothers reported no or relatively slight changes in their and their children’s physical health after displacement. In contrast, six of them clearly stated significant deterioration of perceived mental health (Table 2): “*If you do not take the stress level into account, the health is normal. Fears have still not gone away. I have even asked for psychological help, but the fears are coming back in a month after therapy. Except for this, everything else is fine*” (respondent 3). Two respondents reported severe deterioration of their perceived mental health, including conditions such as stress, depression, anxiety, and constant fear.

### 3.2. Vaccination

General lack of routine child vaccinations after displacement was observed in our respondents. The common practice was that women taking their children for postnatal checkups and immunization found out that there were no vaccines in stock. The reasons seemed to be mostly associated with a lack/rupture of stocks due to the poor economic state of the country following the conflict. The process of waiting for vaccines could take from one month to more than a year, and was consistently reported: “*My child has not received a single vaccine, since they were not available. We missed an opportunity to get vaccination when it was there for one week, and now they told us to wait for another year*” (resp. 4).

### 3.3. Breastfeeding Practices

Seven out of nine respondents have reported no breastfeeding disruptions due to their conflict and displacement experience. All nine interviewees were found to be well-informed about breastfeeding duration and health benefits it brings to a child, with duration of exclusive breastfeeding of six months (as per WHO guidelines) and with solid food introduction after. They also reported having received advice on breastfeeding from the health care personnel: “*I had been breastfeeding before the seventh month. Then, I slowly started introducing semi-solid food, but I’m still breastfeeding*” (resp. 4). Even more, breastfeeding was perceived by six out of nine IDP mothers as a strategy to save money on buying food for children, which is considered a financial coping strategy. 

### 3.4. Nutrition

Significant changes in diet after displacement were reported (Table 2). Due to lower income while displaced, all respondents reported to be forced to cut consumption of meat, fish, fruits and vegetables to almost null: “*Me and my mother, we eat only pasta and grains. Regarding fruits, we give one apple per day to each child, we cannot afford more. We cannot afford meat at all so we don’t eat it*” (resp. 8); “*We mostly use the storages of grains and macaroni we have received as humanitarian help. I cannot even afford to buy a small piece of fish for my child because it is too expensive for us, even though our doctor guided us to do so*” (resp. 4).

### 3.5. Access to Health Care Services in Home Towns

Five respondents out of nine were pregnant at the moment of displacement, and four got pregnant after displacement. All those five respondents who were pregnant reported the destruction of health facilities and infrastructure in their home towns, due to which they missed the obligatory medical check-ups and tests during pregnancy: “…*Back then, the pharmacies were closed, the shops were damaged. I went to my female (gynaecological) consultation to get some paper (certificate) with my husband, and we actually thought that we would never get back alive because of the explosions and fights. I was not able to do the required medical tests*” (resp. 8).

### 3.6. Registration at Health Care Centres in the Place of Displacement

Respondents typically reported seeking a health care center at their new place of living by choosing the closest one to their location and applying for registration. Consequently, our sample of respondents did not reveal any problem of restricted access to health care centers at their displacement area. 

### 3.7. Quality of Health Care

The lack of resources at health care centers and maternity hospitals accessible to IDPs was consistently reported. In particular, this applied to the conditions of stay at maternity hospitals: problems with electricity and running water, poor level of repairs, limited variety in hospital foods, and absence of heating and basic sanitation facilities were common. Three respondents reported that medical personnel were constantly overwhelmed with the flow of patients, which also caused a lack of attention to the patients in need.

### 3.8. Attitudes of the Host Population in Health Care Organizations

The respondents mostly reported to having been well treated in health care centers as well as in maternity hospitals (Table 2): “*When they (health care personnel) knew that we came from Donetsk, they wanted to hug me, they were so caring..... I also found out that in normal practice one has to pay some money annually for female (gynaecological) consultations, but they have not even told me about that. The same was the situation in the maternity hospital; they were really caring once they knew I came from Donetsk*” (resp. 6). 

However, certain cases of health care professionals asking for bribes once knowing about IDP status were also reported. Three respondents noticed problems of enquiries for bribes and the reluctance of receiving health care services without the document of IDP registration from the Ministry of Social Policy in Ukraine having been identified: “*I asked the health care center in Kyiv to register me there. They agreed, but they also asked for a ‘voluntary financial contribution’ amounting to UAN 500 (EUR 20)*” (resp. 1); “*In the health care center for adults there they did not treat me well, they were rude. I had otitis, and they denied helping me unless I paid a bribe*” (resp. 5). It should be noted that these amounts are much lower than the other social payments provided by Ukrainian state.

### 3.9. Household Income Status

Social payments constitute the major source of income for the IDP households. These payments are low and not even sufficient to cover the basic family needs (Table 2): “*The social payments we receive (most on a monthly basis) are those IDPs are entitled for (EUR 32.4 per child and EUR 16.2 per adult), and the state support for the youngest child. The latter is a one-time paid sum of UAN 10,000 (EUR 366.3), and UAN 800 (EUR 32.2) per month until the child reaches three years of age. Considering that we have to pay for rent and utilities, this is not enough*” (resp. 3).

Many also expressed frustration with bureaucracy (long and exhausting paper work while applying for social help) and delays faced when applying for social protection benefits: “*Since my husband is not a Ukrainian citizen, they did not give us the financial support for IDPs and denied us. I tried to dispute it, but they did not want to negotiate*” (resp. 5); “*Other than that, there are long delays with social help, for example, our last payment was delayed for four months because of bureaucracy*” (resp. 4).

### 3.10. Financial Coping Strategies

Applying for humanitarian assistance from NGOs and relief organizations, the use of previous savings and cutting consumption, or purchasing of second-hand goods for children were most common coping strategies. Some respondents noted that they managed to move their clothes and utensils from their home residences to the new place of living, which allowed them to allocate the largest part of their income to purchase food and required goods for children. An important role of community support should also be noted: the most informed respondents created groups in social networks so as to share information about humanitarian assistance, possible promotions, privileges, etc. Community support has also been observed in relation to access to health care services, where IDPs helped with advice and referral to medical doctors.

## 4. Discussion

In this study, we explored IDP mothers’ perceived health and that of their young children, as well as barriers faced in access and delivery of healthcare, child care practices, nutrition security, and poverty. To our knowledge, this is the first attempt to understand maternal and child health of conflict-displaced populations in Ukraine. Our study reveals vulnerabilities that are inherent to the country, such as a weak healthcare system prone to bribes, but also other important consequences that are war related, such as a rupture of vaccine stocks, and halted or delayed vaccinations in children. 

As noted by Rabkin et al. [26], most health services to displaced populations should preferably be provided in dispersed settings within the hosting communities, rather than in traditional refugee camps (or, in the case of Ukraine, in the places of compact residence). This was also the situation observed in Ukraine, where IDPs were not restricted from using health care services within the hosting communities. Therefore, in our setting, the quality of health care provided to IDP mothers and children cannot be viewed separately from the health care system of the country in general, which is low-resourced and poorly financed [27]. Low quality and funding shortages have also been evidenced by the latest Health Systems in Transition report (2015) by WHO [13]. The country’s corrupt health care system has been identified, which makes it more difficult to access health care services for displaced. This was also observed in the conflict in Bosnia [28].

Huge disruptions of vaccine procurement in Ukraine are of clear concern as they could, now or in the near future, generate disease outbreaks. This is a concern for the health of young children, as they are particularly vulnerable to vaccine-preventable diseases such as pertussis and measles. This applies not only to IDPs, but also to the general population. Significant declines in vaccination coverage started in 2014, which have been evidenced by joint WHO and UNICEF estimates [10].

The relative poverty of IDP families described in our study could in the long term also have an impact on maternal and child health. For example, the findings by Lindo [29] and Mashal et al. [30] confirm that parental job losses caused by displacement have significant negative effects on infant health, among other reasons. Moreover, they reduce birth weights on average by 4.5 percent as proven by other research [29]. 

Our study found that the destruction of health care infrastructure in the occupied areas deprived pregnant women of quality health care for several months. This corresponds to previous research by Akol et al. [11], which suggests that the lack of antenatal and perinatal care in war increases the risk of anemia, malnutrition, and concomitant disease. Our study has not identified malnutrition, but has rather found unbalanced nutrition practices, the negative health effects of which tend to appear later and could thus not be felt or measured at this point of research. Another explanation for the findings is that, before 2014, the Ukraine was a food surplus nation and food availability was not a concern [31]. Despite evidence from the Bosnian conflict showing that breastfeeding deteriorates during humanitarian emergencies, when children need it most [32], our study did not find this deterioration. This has a positive health effect, since breastfeeding has been shown to be the most effective way of providing nutrients in humanitarian settings, even in emergency situations where the nutrition status of the mothers is affected [19,33]. These findings, however, can be explained by the small sample size and might not reflect the general tendency. For the future research, it is recommended to look at cross-sectional household surveys, as was done by Andersson et al. [32] for the Bosnian conflict in 1994–1995.

It is worth discussing that mothers’ perceptions of their own physical health or that of their children was not aligned with their reporting of nutritional dietary patterns or that of their children. Given the low consumption of vegetables, meat, and fish, a high prevalence of malnutrition could be normative in these settings. This trend has also been observed in food security assessment conducted by World Food Program (WFP) in April–May 2016 in conflict-affected and bordering areas of Ukraine. WFP found that around 19% of all interviewed households in the area were food insecure, with food consumption levels being more critical among female headed households [34]. 

Food support is one of the most common sources of humanitarian assistance from local and international NGOs and relief organizations. However, the food assistance packages, as reported by respondents, usually consist of foods of low nutritional value like potatoes, macaroni, and grains, as reported by respondents. While they help prevent starvation, they do not contribute to a healthy diet, which is especially needed for child development [35].

It is widely known that suboptimal food dietary intake can cause micronutrient deficiencies [11], which often are not easily perceived by mothers. The same applies to children’s body weight [11]. For the sake of child malnutrition identification, future research should monitor nutritional status of children and their dietary diversity, at least in those under two, and their mothers [35]. The data obtained in this way would allow preventing early malnutrition and design short-term interventions for the treatment of severe malnutrition. Some experiences of these interventions have been reported in conflict settings such as Guinea Bissau [35] or Uganda [36].

Significant deterioration in self-reported mental health of mothers is consistent with findings from a study by Priebe et al. [37], in which they examined the effects of the Balkan war on mental health of mothers and children. They found that both mothers and children showed high levels of post-traumatic stress reactions and increased levels of depression and anxiety. This impact was also evidenced by Panter-Brick et al. [33,38], as well as in the ongoing war in Syria [19]. Our study found reliance on social and community support as one of the coping mechanisms of Ukrainian IDP mothers. Similar mechanisms were also found in other post-emergency settings like Thailand [39] and the Asia-Pacific Region in general [40]. Social support has been found to be the key in psychological resilience building in these settings [41]. Strengthening psychological services and social support, either provided by available community health services or contributed by NGOs, is thus urgently needed in these settings. 

Our study is not exempt of limitations. It should be mentioned the conflict setting made it more difficult to get the larger sample. Therefore, the generalizability of findings is limited. The use of Skype and Viber for interview, technologies which are not available to everyone in Ukraine, may have generated additional exclusions from our sample of mothers, favouring those of higher socio-economic status. The study was thus unable to identify the most marginalized and poor IDPs, who presumably suffer from displacement the most. Other than that, our study could not grasp respondents residing in the places of collective residence/camps, where the health status and the prevalence of e.g., infectious diseases might be worse than those in the population residing within hosting communities [42]. On the other hand, the population residing in places of collective residence communities may have better access to health services [43]. 

Moreover, the use of Skype and Viber limit the personal interaction between the interviewer and interviewee, so the level of trust might be questioned and the control of the interview environment is reduced. The method of Skype/Viber-assisted interviews has been selected due to the absence of possibility to physically travel to Ukraine due to security concerns and limited financial resources.

On the plus side, Skype-mediated interviews can offer more convenient conditions for participants, higher flexibility of setting interview timing, and the possibility to collect non-verbal information (e.g., body language) when the camera is activated [21]. Other than that, evidence identified direct and indirect financial savings when Skype was used in health care research [44]. Also, the use of these technologies expanded our geographical coverage and reduced our research costs. 

Another limitation was that, since the respondents were reached via NGOs, they could possibly identify the interviewer as a provider. This could be described as a limitation of the type of information the interviewer gets, since respondents might tend to emphasize the needs they have, thinking that the interviewer can provide it [21].

## 5. Conclusions

Our study findings highlight the difficulties of the IDP experiences of mothers and their young children in Ukraine. Despite most mothers reporting their physical health and that of their children as good, a pattern of psychological distress was observed among the respondents. Income disruption linked to displacement and insufficient social aid to replace income loss was a common issue faced by most study participants. Overall, mothers affected by income poverty secured housing first and tried to cope by reducing expenses in clothing and utensils, but more importantly by reducing their consumption of fruits, vegetables, meat, fish, and other nutritious foods to almost null. Although uncommon in settings disrupted by displacement, all the interviewed mothers breastfed their children a minimum of six months and lactation was used consciously as a financial coping strategy in these times of food scarcity, so that mothers willingly prolonged it even after six months with the introduction of solid foods. Humanitarian aid by local and international NGOs as well as relief organizations was also noted as relevant by the interviewees. 

More studies are recommended to reinforce the evidence base for policy action in Ukraine, but urgent reforms should not be delayed. It is also recommended to address such themes as sexual and reproductive health and sexual and gender-based violence. Lastly, further research should also allow hearing the voices of the least privileged populations. Based on our findings, we suggest the following recommendations. In the short term, we first recommend restoration of vaccines procurement and distribution by the Ukrainian state to limit the risk of a foreseeable public health crisis in the country. Second, we recommend the provision of psychological support to mothers. Third, it is also advised to launch an educational program on adequate but affordable dietary intake for IDP mothers in order to address inadequate dietary practices. In the mid-term, an augmentation of the IDP allowances and improvement of the bureaucratic process to receive them would be of great value.

## Figures and Tables

**Table 1:** Selected socio-demographic characteristics of the interviewed IDP mothers.

Variable Name	Outcome	Number of Participants
Place of origin	Donetsk city and region	6
	Luhansk city and region	3
Place of displacement *	Kyiv (the capital)	2
	Kyiv region	3
	Eastern Ukraine (adjacent regions)	2
	Central Ukraine	2
Mother age (year)	below 30	2
	30–35	3
	over 35	4
Mother occupation status	Currently employed	3
	Currently unemployed	6
Presence of other dependents (older children, older relatives)	Yes	5
	No	4
Number of children	1	4
	2	3
	3	2
The financial support is provided by a husband/father of a child	Yes	6
	No	3

* As reported during data collection, 1 May–31 June 2016.

**Table 2:** Summary of self-reported health and well-being status.

Variable Name	Outcome	Number of Participants
Perceived physical health *	good	7
	average	2
Perceived mental health	remained stable	3
	deteriorated	4
	severely deteriorated (reported depression, anxiety)	2
Mother’s assessment of child health	good	9
Reported mistreatment in health care centers due to IDP status	yes	3
	no	6
Dietary intake **	sufficient	2
	unbalanced	7
Household income ***	poor	5
	average	2
	high	2

* “good” equals to 80 and higher score of perceived health; “average” equals to 65–80 score. No scores lower than 65 were reported; ** “not balanced” is used when nutrition practices imply inadequate dietary intake, when IDP mothers report they significantly cut meat, fish, fruit, and vegetables and instead ate cheaper products such as pasta, bread, and cereals; *** classified as per the author’s analysis.

## Appendix A. Interview Guide

**Target group**: Internally displaced mothers with children under two years old

**Mode**: In-depth and semi-structured interviews

**Demographic Information**

(1) Age;
(2) Place of origin;
(3) Current location;
(4) When did you give birth?
(5) Previous and current occupation;
(6) Please, tell about your family composition. Who do you live with now? Do you have dependents (other children, older relatives)?

**Military and Displacement experience**

(1) What was the main reason for leaving your home?
(2) Can you describe your displacement experience? Which journey did you undertake to the current place of living? How did you choose the current place of living?
(3) Please, describe where do you live now? How would you describe you living conditions?

**Health Status of mother and child**

(1) On a scale from 0 to 100, can you estimate your current wellbeing, after displacement?
(2) On a scale from 0 to 100, can you estimate the current wellbeing of your younger child after displacement?
(3) If you or your child have or used to have any problems with health during or due to the displacement, which health problems were those?

**Health care services**

(1) Have you used health services in your current location and for which purposes?
(2) What do you think about the quality of health care received in the new place of living?
  - in the maternity hospital (when giving a birth);
  - in the health care center where you are registered now, and from where you get pediatric and maternal health services (after childbirth till now).
(3) Could you describe how well have you been treated there? Can you recall any unpleasant situation related to your IDP status in receiving health care services (e.g., unfair/rude treatment, enquiry for bribes, and denial of health care services provision)?
(4) In your opinion, what is needed to improve availability and quality of services for IDP women and children?
(5) In your new place of living, how did you know about a health care center to visit? Is it easily accessible in terms of distance? How easy is that to get appointment with a health care specialist when needed? How affordable are the health services for you in terms of money?
(6) Before, during, and after pregnancy, have you received enough attention from medical personnel in your new place of living once at the facility? Have you made 4 obligatory visits to an MD before childbirth? Have you received follow-up visits of therapists (e.g., midwives) after childbirth?
(7) Where did you give birth (hospital, home or else)? How/why did you select this location? If it was in the hospital, how long have you been staying there after childbirth?
(8) Has your child been vaccinated? Which vaccines has your child received?
(9) In your new place of living, do you feel that you have been treated well in the hospital?

**Lactation and nutrition**:

(1) How do you store and cook your food? Do you have enough utensils to cook?
(2) Did you breastfeed your child? If yes, for how long? Did you have an immediate contact with your child after childbirth?? If you breastfed, when did you start to change exclusive breastfeeding to the introduction of solid, semi-solid, or soft foods?
(3) Have you received advice on how to breastfeed from your medical specialist (midwife or other)?
(4) Could you describe your weekly ration during and after your pregnancy? If semi-solid and solid foods are already introduced, could you describe weekly ration of your child?

**Financial situation**

(1) Could you please describe your sources of income?
(2) Have you been registered for maternal leave payment (explanation: the lump-sum allowance paid once and keeping the workplace)? If yes, did you manage to obtain this sum, and how long the application process takes?
(3) Generally, do you receive any financial support from the government? If yes, which amount?
(4) Do you receive enough help for caring for a child from your partner/husband/family? If not enough care is received, which are the reasons (cultural norms/conflict/displacement)
(5) From a financial perspective, does your current economic status allow you to purchase:
  - groceries for you and your child (sufficient amount and quality)?
  - required medications for you and your child?
  - required health care services and procedures?
  - other goods for your child (hygiene-related, toys, etc.)
(6) Are there any other issues that you think of, related to this?
(7) If you do not meet basic needs for yourself and your children, how do you manage the situation?
